# Household Structure and Older Persons

**DOI:** 10.1093/geroni/igab046.1926

**Published:** 2021-12-17

**Authors:** Sainan Zhang, Sandile Simelane, Tapiwa Jhamba, Rachel Snow

**Affiliations:** 1 UNFPA, New York, New York, United States; 2 United Nations Population Fund, New York, New York, United States

## Abstract

This research explores the life circumstances of older persons (aged 60 years and above), focusing on the sociodemographic and socioeconomic conditions of those who live alone. We situate the living arrangements of older persons within the global context of changing household structures in 76 countries from all regions of the world. Older persons who live alone are among those most likely to need governmental and other forms of social support. The analysis presented here is crucial for supporting policy responses to the needs of older persons, including the special attention they require during the current COVID-19 crisis. It also supports the operationalization of the Madrid International Plan of Action on Ageing (MIPAA)(United Nations, 2002), the realization of United Nations Principles for Older Persons (United Nations, 1991), and the broader framework of the Programme of Action of the International Conference on Population and Development(ICPA-POA).

